# Antimicrobial activity of ProRoot MTA in contact with blood

**DOI:** 10.1038/srep41359

**Published:** 2017-01-27

**Authors:** C. Farrugia, P. Baca, J. Camilleri, M. T. Arias Moliz

**Affiliations:** 1 Department of Restorative Dentistry, Faculty of Dental Surgery, University of Malta, Msida, Malta; 2 Department of Stomatology, Faculty of Dentistry, University of Granada, Campus Cartuja s/n, Colegio Máximo 18071, Granada, Spain; 3 Department of Microbiology, Faculty of Dentistry, University of Granada, Campus Cartuja s/n, Colegio Máximo 18071, Granada, Spain

## Abstract

Dental materials based on Portland cement, which is used in the construction industry have gained popularity for clinical use due to their hydraulic properties, the interaction with tooth tissue and their antimicrobial properties. The antimicrobial properties are optimal *in vitro*. However in clinical use contact with blood may affect the antimicrobial properties. This study aims to assess whether antimicrobial properties of the Portland cement-based dental cements such as mineral trioxide aggregate (MTA) are also affected by contact with blood present in clinical situations. ProRoot MTA, a Portland cement-based dental cement was characterized following contact with water, or heparinized blood after 1 day and 7 days aging. The antimicrobial activity under the mentioned conditions was assessed using 3 antimicrobial tests: agar diffusion test, direct contact test and intratubular infection test. MTA in contact with blood was severely discoloured, exhibited an additional phosphorus peak in elemental analysis, no calcium hydroxide peaks and no areas of bacterial inhibition growth in the agar diffusion test were demonstrated. ProRoot MTA showed limited antimicrobial activity, in both the direct contact test and intratubular infection test. When aged in water ProRoot MTA showed higher antimicrobial activity than when aged in blood. Antimicrobial activity reduced significantly after 7 days. Further assessment is required to investigate behaviour in clinical situations.

The main challenge faced by dental cements in clinical use is the moisture and the presence of bacteria. In fact the ideal dental cement should improve its characteristics and properties in the presence of moisture and also be antibacterial. The popularity with Portland cement-based materials used as dental cements for root-end filling after apicectomy and for repair of root perforations is their hydraulic nature^1^ and their antimicrobial activity^2^. Elimination of microorganisms from the root canal system is one of the main goals of endodontic treatment. However, the treatment reduces but does not eliminate all microorganisms and viable bacteria often remain inside dentinal tubules and root canal irregularities^3^. Thus, the use of root filling materials with antimicrobial properties is important. The first Portland cement-based material used as a dental cement was mineral trioxide aggregate (MTA) which is a mixture of Portland cement and bismuth oxide added for radiopacity. When MTA is used as a root-end filling it is claimed to form a bacterial-resistant barrier^2^ that has been attributed to the presence of calcium hydroxide in the set materials^4^.

Although Portland cement-based materials are hydraulic, contact with blood and tissue fluids resulted in lack of material hydration^5^,^6^ and deterioration of physical properties^7^. The calcium hydroxide, which is a product of material hydration was not present when MTA was placed in contact with blood^5^. Since the antimicrobial properties of MTA are attributed to the alkaline pH resulting for the formation of calcium hydroxide, it can be postulated that the antimicrobial activity of the material will reduce in the presence of blood. Furthermore MTA discolouration was noted in the presence of blood^8^,^9^. The aim of this research was to assess the chemical changes, discoloration potential and antimicrobial activity of ProRoot MTA after being exposed to blood, which is the environment found clinically during the material’s multipurpose use.

## Results

### Material characterization

**Figure 1:**
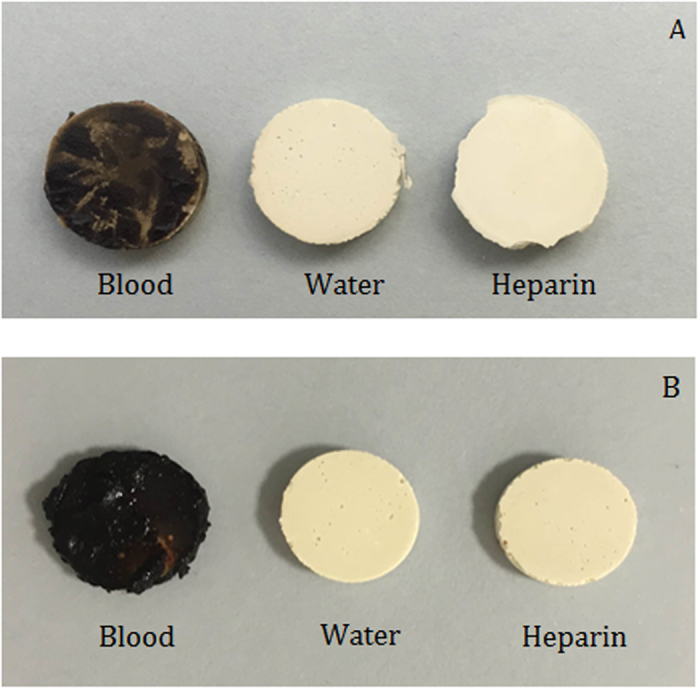
Material discs after (**A**) 1 day and (**B**) 7 days in contact with the test solutions.

**Figure 2:**
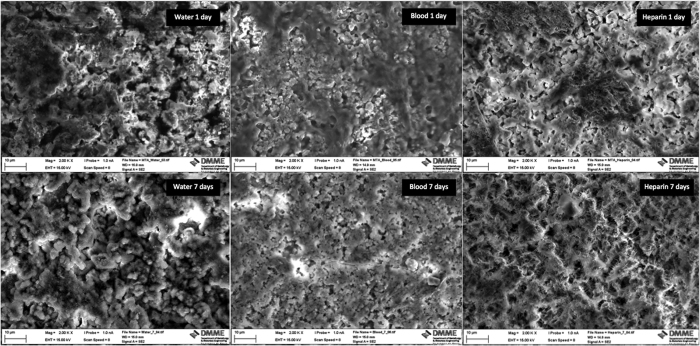
Scanning electron micrographs of ProRoot MTA in contact with water, blood and heparin for 1 day and 7 days.

**Figure 3:**
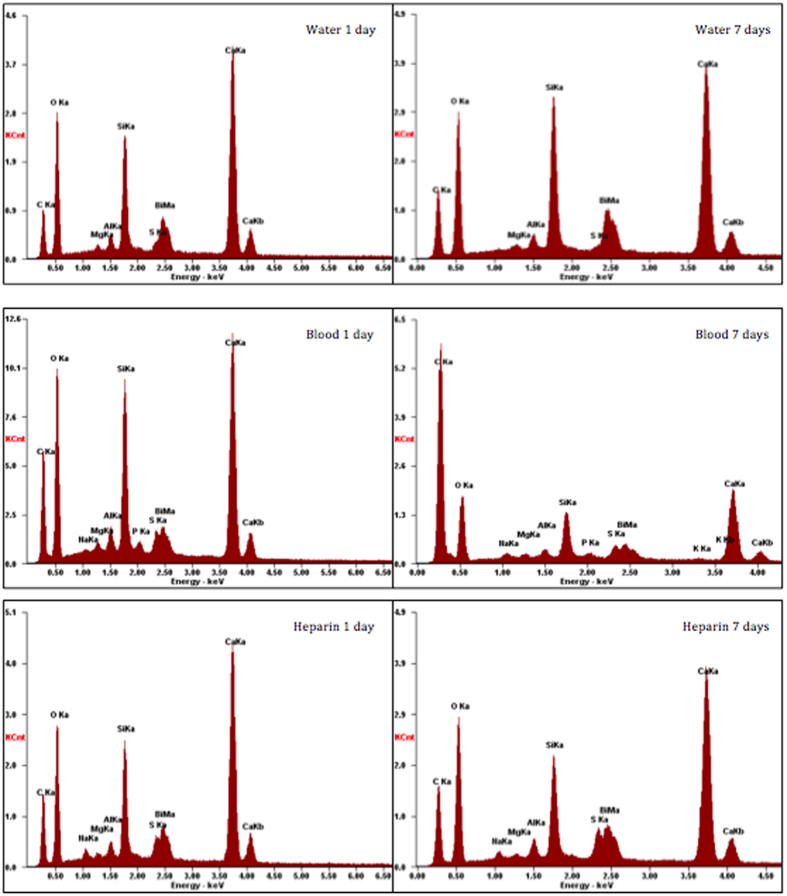
Energy dispersive spectroscopic plots of ProRoot MTA in contact with water, blood and heparin for 1 day and 7 days.

All the materials that were immersed in blood exhibited a black discoloration (Fig. 1). The set materials in contact with the various solutions were characterized by scanning electron microscopy (SEM), energy dispersive spectroscopy (EDS) and X-ray diffraction (XRD) analyses. Thus the three methods were used simultaneously to ensure a full characterization was carried out. The scanning electron microscopy showed the surface microstructural changes (Fig. 2) with a rough surface morphology for the different environments. The MTA in contact with heparin for 7 days exhibited cement particles with elongated processes showing cement reaction foci (Fig. 2). The EDS analyses (Fig. 3) demonstrated the changes in elemental composition of the materials in contact with the different solutions. Peaks for calcium, silicon, oxygen, bismuth, aluminium and sulphur were present in all tested materials. In addition the ProRoot MTA exposed to blood exhibited an additional phosphorus peak at both day 1 and day 7 (Fig. 3). The MTA exposed to both blood and heparin had an additional sodium peak discerned in the EDS analysis (Fig. 3).

**Figure 4:**
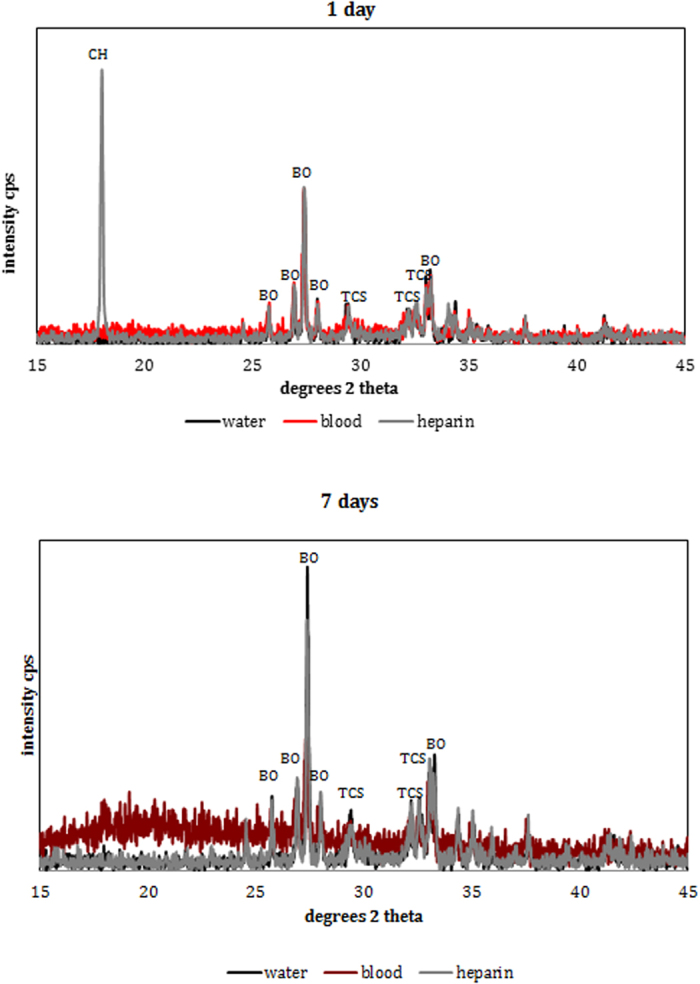
X-ray diffraction scans of ProRoot MTA in contact with different solutions for 1 day and 7 days.

The XRD plots (Fig. 4) showed the phase changes. For 1-day immersion, there were identical peaks for both ProRoot MTA immersed in water and in blood (Fig. 4). The material was composed of tricalcium silicate and bismuth oxide. The ProRoot MTA in contact with heparin showed an additional peak of calcium hydroxide at 18° 2θ which is synonymous for calcium hydroxide presence (Fig. 4). After 7 days of exposure to the solutions the MTA in contact with water and heparin exhibited similar peaks for tricalcium silicate and bismuth oxide (Fig. 4). The MTA exposed to blood exhibited only the bismuth oxide peaks and a general wavy baseline showing the presence of an amorphous phase (Fig. 4).

### Antimicrobial activity analysis

The antimicrobial activity of the materials was analyzed using three methods: the agar diffusion test that determines the antimicrobial activity on the contaminated agar, the direct contact test where the material is in contact with the bacterial suspension and the intratubular infection test that evaluates the antimicrobial activity of the material against infected dentin tubules.

**Figure 5:**
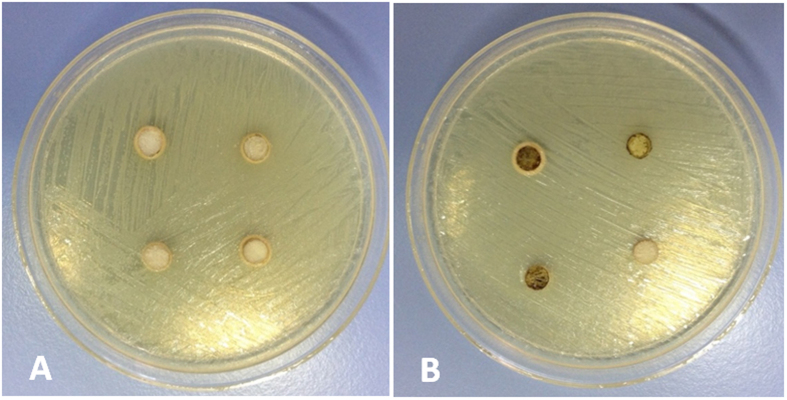
Agar diffusion test images of ProRoot MTA aged in water for 7 days (**A**) and ProRoot MTA aged in blood for 7 days (**B**). Four replicates per plate are observed.

**Figure 6:**
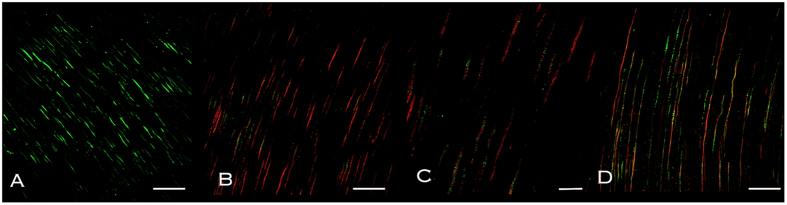
Representative confocal laser scanning microscope images of (**A**) control group, (**B**) ProRoot MTA aged in water and (**D**) ProRoot MTA aged in blood, for 1 day. Bars represent 50 μm. A magnification of the dentinal tubules of the MTA aged in water show cocoidal cells presenting green and red fluorescence (**C**). The bar represents 10 μm.

In the agar diffusion test no inhibition zones were found in any of the study groups. Representative images of the agar diffusion test are shown in Fig. 5. The results of the direct contact test and the intratubular infection test showed that although limited, ProRoot MTA exerted antimicrobial activity, with values over 3 logarithmic units in the direct contact test and under 70% of dead cells in the intratubular infection test. When aged in water for 1 and 7 days, ProRoot MTA showed significantly lower viable counts and higher death percentages than when aged in blood (p < 0.05). After 7 days, the antimicrobial activity was significantly reduced compared with 1 day (p < 0.05), and in the case of ProRoot MTA aged in blood it showed statistical similar values to the control (p > 0.05). Results for direct contact test and intratubular infection test are shown in Tables 1 and 2. Representative CLSM images of the infected dentin in contact with ProRoot MTA are shown in Fig. 6. The dead cells give a green fluorescence while the dead cells are in red.

## Discussion

The antimicrobial properties of mineral trioxide aggregate have been extensively tested in the literature. Most antimicrobial testing has been restricted to uncontaminated and unaged materials, without contact with physiologic solutions and by using simple tests such as the agar diffusion test^10^,^11^. In the current study the materials were also characterized in contact with blood and a separate test with contact with heparin was also carried out to ensure that the use of an anticoagulant did not affect the material properties. The hydration state was correlated to the antimicrobial properties. The antimicrobial properties were tested using standard methods such as agar diffusion test and direct contact test but the intratubular infection test was also carried out. In dental infections most of the bacteria are not in suspension but are biofilms. The root dentine is a very particular substrate and in long standing endodontic infections which, necessitate the use of materials such as MTA the resistant bacteria such as *E. faecalis* are incarcerated in the dentinal tubules thus very difficult to eradicate The intratubular infection test mimics such conditions thus is the best test to use for testing the antimicrobial properties of endodontic materials.

A dental cement should not be adversely affected by blood or other physiological solutions. Some studies have noted changes in MTA’s properties when in contact with blood^7^,^12^,^13^. Therefore, this study aimed at assessing the changes occurring in MTA following prolonged contact with blood and whether the blood affects the material’s antimicrobial properties.

ProRoot MTA was selected since it is the first commercial MTA to be launched and one of the most commonly studied MTA materials on the market, tested by several other researchers for its interaction with blood^2^,^5^,^7^. Whole human blood was chosen to replicate clinical conditions in humans as has been performed in other studies^7^. Heparin, known for its use in medicine^14^, was used to prevent blood from coagulating during the exposure times chosen. The effect of heparin on cement hydration has never been investigated although it has been used in previous research on MTA. Thus an additional group with ProRoot MTA immersed in heparin was added to investigate the effects of heparin on the MTA. The heparin accelerated cement hydration in the early ages as indicated by the calcium hydroxide peak shown in the XRD scans after 1-day exposure. The use of heparin is also responsible for the sodium peak found in the EDS analysis.

Although heparin clearly accelerated the cement hydration, the addition of heparin to the blood did not enhance material hydration and no calcium hydroxide was formed over the 7-day immersion period. Thus the blood further restricted the material hydration as verified in previous research^7^. The presence of phosphates as shown in the EDS analysis and the amorphous nature of the precipitate on the material surface indicate the reaction of MTA with blood very likely forming amorphous calcium phosphate phases which are the precursors to apatite that have been reported as being deposited on the MTA surface in the presence of simulated tissue fluids^15^,^16^. However since the deposit was amorphous the exact chemical nature could not be verified using X-ray diffraction analysis. The formation of a phosphate phase had never been reported with MTA in the presence of blood.

Similar to other studies^8^,^13^ colour changes were noted on all material samples exposed to blood (Fig. 1), even after rinsing with sterile water, suggesting that blood infiltrated in the material’s surface affecting the material chemistry.

Although endodontic infections are polymicrobial^17^, a single species culture of *E. faecalis* was selected for various reasons: it is the most frequent strain in treatment-resistant cases^18^,^19^,^20^, it is commonly used to assess the antimicrobial properties of materials against endodontic infections as reported previously^21^ and it was confirmed in a pilot study (data not shown) that it resists centrifugation. Resistance to centrifugation is important because many bacterial species involved in endodontic infections are obligate anaerobic bacteria^22^ that would not survive the centrifugation process due to air flow and forces. The use of multiple tests to assess the antimicrobial properties of MTA ensured data reproducibility. The agar diffusion test is a simple test most commonly used for assessment of materials, including calcium silicates^12^. Its simplicity has made this methodology very popular but it relies on the solubility of the materials in question. This method is not quantitative^23^ and does not distinguish between bacteriostatic and bactericidal effects^11^. The direct contact test was used as a more quantitative and reproducible method to simulate the contact of the test microorganisms with the materials and measure the bacterial growth in presence of them^24^. The selected time of 1 hour allows a direct contact of the bacteria to the material. It is possible that some bacteria adhere to the surface within this time period. However, it would be an initial stage of interaction so that bacteria are recovered easily thus reducing the alteration of the results^25^,^26^. Although ideally there should be a challenge concentration for endodontic cements, no threshold has been set^27^. Instead, the results are normally compared to the positive control, which in this case is the bacteria in contact with the polystyrene surface of the microtiter plate. In order to assess in further detail the antimicrobial activity of the materials against bacteria inside dentinal tubules, the intratubular infection test was carried out. This method provides for an artificial and reproducible infection of dentin tubules by centrifugation^21^,^24^,^28^, where as the use of CLSM and the Live/Dead staining allows the viability of the residual bacteria inside dentin tubules to be evaluated. The scanning is performed inside dentin structure, 5–10 μm from the subsurface level of the dentin allowing quantification of undisturbed bacteria inside dentin tubules^24^. Furthermore, the effect of the dentin on the antimicrobial properties of the material is also assessed with this method thus it is more clinically relevant. To date there is no information about the antimicrobial activity of ProRoot MTA against bacteria infected dentin. Testing in these conditions is important as this material is normally placed on infected dentin.

Antimicrobial activity is expected from tricalcium silicate-based materials because of the pH increase resulting from the formation of calcium hydroxide during the hydration reaction^29^. The antimicrobial properties of ProRoot MTA have been evaluated against different bacteria including *E. faecalis* obtaining controversial results. Some authors have reported a limited activity^30^ whereas it was effective in other studies^30^,^31^,^32^,^33^. However, this is the first study to evaluate the effect of different fluids on the antimicrobial properties of a MTA-based material. In the present work, no antimicrobial activity was observed in the ProRoot MTA when in contact with water or blood in the agar diffusion test. This result is in accordance to previous studies^10^,^30^ although no fluids were used to age the materials in the mentioned studies. However, because of the limitations of this technique these results lack reliability. In contrast, in the direct contact test and intratubular infection test some antibacterial action was shown by the ProRoot MTA placed in both water and blood, with samples placed in blood showing significantly less antibacterial activity. Calcium hydroxide has been shown to be ineffective in eliminating *E. faecalis* when used in the root canal, because the buffering capacity of dentin makes it difficult to maintain a high pH in the dentin^34^,^35^. On the other hand, Zhang *et al*.^32^ presented data showing that dentin powder enhances the effect that ProRoot MTA has in elimination of *E. faecalis*^36^. In the present study however, a similar behavior on the antimicrobial properties of the ProRoot MTA in the absence (direct contact test) or presence of dentin (intratubular infection test) were found. As a consequence it has been suggested that the elimination of *E. faecalis* by the MTA in the presence of dentin could be caused by factors other than pH alone, like the ion release^32^; that at the same time could be influenced by the fluid. Further assessment on the role of this ion release would be of value.

In this study, although the reduction achieved by MTA aged in water and blood for 1 day was 1.67 and 0.82 logarithmic units respectively, the percentage of reduction in bacterial counts was 97.30% and 82.64%, respectively. A similar reduction of 81.76% was observed in the material aged in water for 7 days. It is likely that such bacterial reduction is acceptable from a clinical point of view. However, this antimicrobial activity was lost when the ProRoot MTA was in contact with blood for 7 days. The results of the intratubular infection test are consistent with the ones of the direct contact test supporting the lack of activity of the ProRoot MTA after being aged with the blood for 7 days.

This drastic drop in the antimicrobial activity in presence of blood could be explained by different mechanisms. First, the nutrients coming from blood could have reached the bacteria inside dentin tubules through leakage. Second, the biomineralized interfacial layer with tag-like structures at the ProRoot MTA-dentin interface^37^ could have been altered by the presence of blood. Indeed, as found in this study ProRoot MTA has been shown to reduce the formation of calcium hydroxide when in contact with blood^5^ and this could be the main mechanism to explain the reduction in the antimicrobial properties in presence of blood. In a recent study on Endosequence BC RMM putty which is also a tricalcium silicate-based material, it was shown that biomineralization with formation of hydroxyapatite on the material surface was demonstrated in contact with simulated body fluid as reported previously^15^,^38^. However investigation of material retrieved from a surgical site of a failed root-end surgery exhibited the deposition of calcium carbonate^38^. In view of these results, chemical changes in presence of blood and *in vivo* would infer in the material-dentin interface allowing nutrients and bacterial leakage. The biomineralization activity of ProRoot MTA clearly reduced its antimicrobial potential.

## Methods

ProRoot MTA (Dentsply Tulsa Dental Specialities, Tulsa, OK, USA) was mixed at a water to powder ratio of 0.33 following manufacturer’s recommendations. ProRoot MTA was left to set for 24 hours in a humid environment at 37 °C. All the experimental protocols were approved by the ethics committee of the University of Granada, Spain. Human blood was obtained by phlebotomy using a 23-gauge needle from a healthy volunteer member of the research group who gave informed consent in compliance with the “Declaration of Helsinki ethical principles (2000)”^5^,^7^,^9^. The Ethics Committee of the University of Granada also approved the protocol [UGR-438] for the use of human teeth in the intratubular infection test. All methods were performed in accordance with the relevant guidelines and regulations.

For the antimicrobial activity testing 4 study groups were included: Group 1: MTA in contact with sterile water for 1 day; Group 2: MTA in contact with whole heparinized blood (Heparin sodium, 5,000 I.U./ml, Wockhardt, Wrexham, UK) for 1 day; Group 3: MTA in contact with sterile water for 7 days; Group 4: MTA in contact with whole heparinized blood for 7 days. For material characterization two additional groups were included. Group 5: MTA in contact with heparin for 1 day; Group 6: MTA in contact with heparin for 7 days. The Group 5 and 6 were characterized to investigate the effect of heparin on material characteristics but the antimicrobial testing was not performed as this was not clinically relevant.

### Material characterization

Samples of 10 mm diameter and 2 mm thick were placed in either water or heparinized whole blood for 1 and 7 days. The effect of heparin on the material hydration was also assessed by immersing specimens in heparin for both time frames. After 1 and 7 days the specimens were removed from solution and were dried in a vacuum desiccator using silica gel.

#### Sample photography

The materials were photographed against a light grey background to determine color changes visually.

#### Scanning electron microscopy and energy dispersive spectroscopy

The samples were characterised using a scanning electron microscope (SEM) coupled with an energy dispersive spectroscope (EDS). After drying, the specimens were mounted on aluminium stubs, carbon coated and viewed under a scanning electron microscope (Zeiss MERLIN Field Emission SEM, Carl Zeiss NTS GmbH, Oberkochen, Germany). Scanning electron micrographs of the different material microstructural components 2 K X magnification taken in secondary electron mode at a working distance of 15 mm were captured. Energy dispersive spectroscopy of the different phases to investigate the elemental analyses was carried out.

#### X-ray diffraction analysis

ProRoot MTA placed in water, whole heparinized blood and heparin for 1 and 7 days was characterized by X-ray diffraction analysis (XRD) in Bragg Brentano geometry. The diffractometer, a Bruker D8 diffractometer (Bruker Corp., Billerica, MA, USA) used Co Kα radiation (1.79 Å) at 40 mA and 45 kV. The X-ray patterns were acquired in the 2θ (15–45°) with a step of 0.02° and 0.6 seconds per step. The specimens were continuously rotated at 15 revolutions per minute. Phase identification was accomplished using a search-match software utilizing ICDD database (International Centre for Diffraction Data, Newtown Square, PA, USA).

### Antimicrobial activity analysis

Three antimicrobial methods were performed: agar diffusion test, direct contact test and the intratubular infection test. The bacterial strain used was *Enterococcus faecalis* ATCC 29212^18^,^19^. All bacterial suspensions were done in brain-heart infusion (BHI) (Scharlau Chemie S.A., Barcelona, Spain) broth and adjusted using a turbidimeter (Densichek Plus, Biomeriuex, Boston, USA).

#### Agar diffusion test

Twenty four samples 1 mm height and 6 mm diameter were exposed to 1 mL of water or blood for 1 or 7 days according to the groups described before (n = 6).

For the agar diffusion test, a previously described methodology was used^10^. Briefly, 100 μL of a 0.5 McFarland bacterial suspension were spread evenly on BHI agar plates. The plates were then left for 30 minutes at room temperature. The aged samples were rinsed in sterile distilled water, dried on sterile filter paper and then placed on the BHI agar plates. After incubation for 24 hours at 37 °C, microbial inhibition zones were measured with a precision rule. Six replicates per group were tested.

#### Direct contact test

For the direct contact test^4^ an area of established dimensions on one side of the wells of a 96-well microtiter plate (Nunclon Delta Surface; Nunc, Roskilde, Denmark) was coated with an equal amount of MTA using a sterile spatula. Once the material was set, samples were exposed to 220 μl of sterile distilled water or heparinized blood under the conditions mentioned before. After aging, the solution was rinsed off and, with the plate held vertically, a 10-μL aliquot of the bacterial suspension of approximately 1 × 10^7^ colony forming units per milliliter (CFU/mL) was placed on the surface of each sample. Ten microliters of bacterial suspension placed on the wall of uncoated wells without the tested materials served as the positive control. After incubation for 1 hour at 37 °C to ensure direct contact between bacteria and tested materials, 220 μL of sterile BHI was added to each well. The bacterial suspension was mixed for 1 minute in order to recover the bacteria that can have adhered to the material surface, diluted serially and plated on BHI agar plates for viable cell counting. Each group was tested twice, and the procedure was performed in triplicate for a total of 6 replicates per group.

#### Intratubular infection test

**Figure 7:**
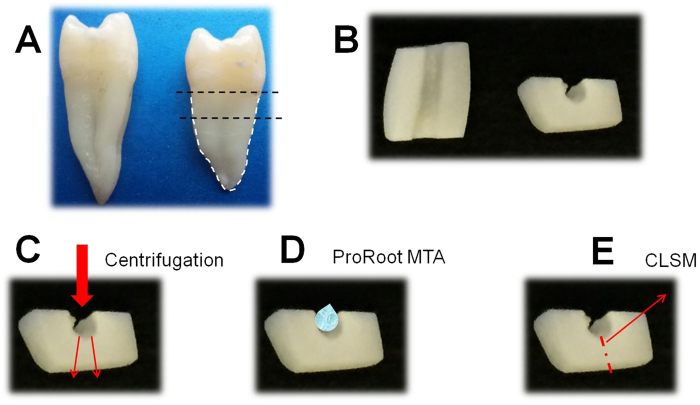
Schematic description of the intratubular infection test: (**A**) cylindrical root segments of 4 mm length, (**B**) root canal samples after sectioning the cylindrical segments into two halves, (**C**) contamination of dentin tubules by centrifugation, (**D**) ProRoot MTA placed on the root canal wall, (**E**) the samples are cut into two halves and a fresh surface is observed under the confocal laser scanning microscopy (CLSM).

For the intratubular infection test a previously described methodology was used^21^,^24^, with slight modifications. Cylindrical root segments of 4 mm length (Fig. 7A) were obtained from 15 non-carious human maxillary premolars by sectioning the root horizontally 1 mm below the cement-enamel junction using an Accuton-50 machine (Struers, Copenhagen, Denmark). Each root canal was enlarged to the size of a Gates Glidden bur #4. The root segments were then sectioned into two halves with a diamond disk (355514220 HP, Edenta AG, Switzerland). After removing the outer cementum the size was adjusted by polishing with 220- to 800-grit SIC papers to obtain 4 × 4 × 2 mm specimens (Fig. 7B). The smear layer formed during preparation of the dentin specimens was removed with 17% EDTA for 10 minutes. Then, the samples were washed with distilled water for 10 minutes and sterilized by autoclave. Each prepared dentin specimen was placed in the upper chamber of a filter tube (VWR International Eurolab SL, Barcelona, Spain) with the canal side up, and gaps with the inner wall of the tube were sealed with flowable composite resin.

For dentin tubule infection, 500 μL of an *E. faecalis* suspension of approximately 1 × 10^7^ CFU/mL was added to the upper chamber of each filter tube with the dentin specimen inside. The tubes were centrifuged at 1400 g, 2000 g, 3600 g, and 5000 g in sequence, twice each for 5 minutes (Fig. 7C). Between the centrifugations, the suspension was replaced with 500 μl of a fresh one. The same procedure was repeated after two days. The samples were incubated for a total of 5 days at 37 °C.

After taking the specimens out of each tube, they were washed with saline solution and randomly divided into the previously described 4 groups (n = 5). The material was placed on the surface of the root canal wall to achieve an approximate thickness of 0.5 mm (Fig. 7D). After setting, the samples were exposed to 220 μL of blood or water for 1 and 7 days at 37 °C. Two positive control groups were also included (n = 5) which consisted of dentin specimens without MTA and fluid that were aged for 1 and 7 days. After aging, the specimens were washed in saline solution twice for 1 minute and vertically cut into two halves through the root canal using an Accuton-50 machine with saline solution as irrigant. Thereafter, they were washed with saline solution, stained and observed under CLSM (Fig. 7E). For dentin disinfection analysis, the Syto-9/Propidium iodide (PI) technique (Live/Dead, Baclight, Invitrogen, Eugene, OR, USA) was used^39^. SYTO-9 is a green-fluorescent stain, labeling both live and dead microorganisms. PI is a red-fluorescent nucleic acid stain and penetrates only the cells with damaged membranes (dead microbes). All the samples were observed using a confocal laser-scanning microscope (Nikon Eclipse Ti-E, Nikon Canada, Mississauga, Canada). Four microscopical confocal volumes from random areas were obtained from each sample (a total of 20 operative fields per group) using a 40 × oil immersion objective, 1 μm step-size and a format of 512 × 512 pixels. Each picture represented an area of 317 × 317 μm. The scanning was performed inside the dentin structure, 5–10 μm from the subsurface level of the dentin in order to obtain the fluorescence of bacteria non-affected by the sectioning procedure. For quantification purposes *bio*image_L software was used^40^. The parameter evaluated in each group was the percentage of red population (dead cells).

### Statistical analysis

Results of the direct contact test were expressed as log_10_ CFU + 1/mL and of the intratubular infection test as percentage of dead cells previously subjecting the data to the Anscombe transformation^41^. An ANOVA test followed by the Tukey post hoc test were used for comparisons among groups at 1 and 7 days and *t*-Student test was used for comparisons between the two aging periods (*p* < 0.05). Statistical analyses were performed by means of SPSS 20.0 software (SPSS Inc, Chicago, IL).

## Conclusions

MTA in contact with blood exhibited discoloration and reduced antimicrobial activity caused by the biomineralization of MTA in the presence of phosphates in the blood. Further research under conditions closer to the clinical situation like longer exposure periods and other tests specially adapted to polymicrobial biofilms are required to confirm these preliminary findings and to ensure long-term clinical performance of MTA and its long term antimicrobial activity.

## Additional Information

**How to cite this article**: Farrugia, C. *et al*. Antimicrobial activity of ProRoot MTA in contact with blood. *Sci. Rep.* **7**, 41359; doi: 10.1038/srep41359 (2017).

**Publisher's note:** Springer Nature remains neutral with regard to jurisdictional claims in published maps and institutional affiliations.

## Figures and Tables

**Table 1:** Viable counts expressed as mean Log_10_ (standard deviation) of *E. faecalis* after being in contact to ProRoot MTA aged in water and blood for 1 and 7 days.

	1 day	7 days
MTA water	3.86 (0.45)^a,1^	4.79 (0.29)^a,2^
MTA blood	4.71 (0.40)^b,1^	5.41 (0.12)^b,2^
Positive control	5.53 (0.31)^c,1^	5.59 (0.21)^b,1^

Read vertically, different superscript letters indicate statistical differences by Tukey test after ANOVA showed significant values.

Read horizontally, the same numbers show differences not statistically significant by *t*-Student test.

**Table 2:** Mean (standard deviation) of the percentage of dead cells inside dentin tubules after exposure to ProRoot MTA aged in water and blood for 1 and 7 days.

	1 day	7 days
MTA water	68.61 (20.48)^a,1^	52.04 (23.68)^a,2^
MTA blood	53.86 (15.35)^b,1^	22.60 (22.48)^b,2^
Positive control	14.20 (16.79)^c,1^	21.61 (14.87)^b,1^

Read vertically, the same letters show differences not statistically significant by Tukey test after ANOVA showed significant values.

Read horizontally, the same numbers show differences not statistically significant by *t*-Student test.
